# Chinese Herbal Formula Huoxiang Zhengqi for Dampness Pattern in Atopic Dermatitis and Diarrhea-Predominant Irritable Bowel Syndrome: Rationale and Design of a Master Protocol

**DOI:** 10.1155/2021/5125568

**Published:** 2021-10-04

**Authors:** Qian Huang, Xiaohui Guo, Meiling Xuan, Wenwei Ouyang, Zehuai Wen

**Affiliations:** ^1^Second Clinical Medical College of Guangzhou University of Chinese Medicine, Guangzhou 510120, China; ^2^Key Unit of Methodology in Clinical Research, Guangdong Provincial Hospital of Chinese Medicine, Guangzhou 510120, China; ^3^State Key Laboratory of Dampness Syndrome of Chinese Medicine, Second Affiliated Hospital of Guangzhou University of Chinese Medicine, Guangzhou 510120, China; ^4^Science and Technology Innovation Center, Guangzhou University of Chinese Medicine, Guangzhou 510405, China

## Abstract

*Introduction*. Atopic dermatitis (AD) and diarrhea-predominant irritable bowel syndrome (IBS-D) are two recurrent diseases with limited effective treatments. In Chinese Medicine (CM) theory, they may share dampness pattern as the same dominant pathogenesis at a certain stage and, thus, can be treated with the same method. While Chinese herbal formula Huoxiang Zhengqi (HXZQ) has been reported as an effective dampness-resolving therapy for both AD and IBS-D, further high-quality clinical studies are still needed. In addition, HXZQ lacks accurate clinical positioning based on CM patterns. Therefore, we utilize a master protocol design to evaluate HXZQ for dampness pattern simultaneously in AD and IBS-D, with the aim of identifying the pattern-defined population of HXZQ. *Methods and Analysis*. This master protocol design includes two randomized controlled trials (RCTs) and a real-world observational study. Based on two registry cohorts of AD and IBS-D, patients with dampness pattern will be enrolled in the RCTs to receive either HXZQ oral liquid or a placebo for 4 weeks and then will be followed up for another 4 weeks, while patients with nondampness pattern will constitute the observational study and experience a 12-week follow-up. A total of 678 AD patients and 322 IBS-D patients will be recruited from 14 hospitals in China over a 3-year period. The eczema area and severity index (EASI) and the proportion of responders for adequate relief (AR) are the primary outcomes in AD and IBS-D, respectively. Analysis will be undertaken separately in each substudy, and then an overall analysis combining multiple subgroups will be performed to comprehensively investigate the effect of HXZQ. *Discussion*. This study will provide high-quality efficacy evidence of HXZQ for AD and IBS-D patients and give an example of postmarketing evaluation for CM products under the pattern dominating different disease research model. The study is registered with ChiCTR1900026700 and ChiCTR1900026837.

## 1. Introduction

Atopic dermatitis (AD) is an inflammatory skin disorder characterized by intense itching and recurrent eczematous lesions. The prevalence of AD has been increased worldwide for decades [1], and it was estimated to be 4.6% among adults in China [2]. Conventional medications for AD including topical corticosteroids and calcineurin inhibitors are often associated with unwanted side effects [3], while Chinese medicine (CM) has been reported to be well tolerated and has advantages in improving health-related quality of life in AD patients [4, 5]. Therefore, CM has been a common therapeutic option for AD in China.

Irritable bowel syndrome (IBS) is a group of functional gastrointestinal disorders characterized by abdominal pain that may be associated with changes in bowel habits [6]. It heavily diminishes patients' quality of life and increases healthcare costs. The pathophysiological mechanism of IBS remains unknown, so current available treatments are limited [7]. In China, diarrhea-predominant IBS (IBS-D) is the most common subtype of IBS, and the initial therapies for IBS-D involving antidiarrheal, dietary, and lifestyle adjustments are often ineffective [8]. Fortunately, CM with a long history of treating functional bowel disorders provides a promising therapy choice [9, 10].

In CM theory, AD and IBS-D are two different diseases but can share dampness pattern (Shi Zheng) as the same dominant pathogenesis at a certain stage [11, 12]. Dampness pattern is one of the most common CM pathological mechanisms and occurs in many diseases. The clinical manifestations of dampness pattern include mental fatigue, heavy sensation in the limbs and body, and sticky or greasy tongue coating. Moreover, following the CM therapeutic principle of “treating different diseases with the same therapy,” AD and IBS-D can be treated with the same dampness-resolving method if they are both manifested as dampness pattern. Chinese herbal formula Huoxiang Zhengqi (HXZQ) is a classic dampness-resolving prescription that has been widely used since the early twelfth century. Clinical studies have suggested that it may be a safe and effective treatment for both AD [13, 14] and IBS-D [15, 16]. However, given the inadequate design of these studies, the benefits of HXZQ remain unclear in these two diseases.

HXZQ is composed of Herba Pogostemonis (Huoxiang), Pericarpium Arecae (Dafupi), Perillae (Zisu), Rhizoma Pinelliae (Banxia), Pericarpium Citri Reticulatae (Chenpi), Cortex Magnoliae Officinalis (Houpo), Radix Angelicae Dahuricae (Baizhi), Poria (Fuling), Rhizoma Atractylodis Macrocephalae (Baizhu), Radix Platycodonis (Jiegeng), and Radix Glycyrrhizae (Gancao). Experiments have revealed that it could resolve dampness pattern in rat models through multiple approaches: normalizing gastrointestinal dysfunction [17], improving immune and metabolic functions [18], and regulating gut microbiota disturbance [19]. Currently, HXZQ is an over-the-counter (OTC) medication and has various dosage forms such as oral liquids, capsules, and pills [20]. Despite its rich history of human use, HXZQ still requires high-quality evidence of postmarketing evaluation when used as a Chinese patent medicine. In addition, it also lacks accurate clinical positioning based on CM patterns.

Hence, we utilize a master protocol comprising two randomized controlled trials (RCTs) and a real-world observational study to evaluate HXZQ oral liquid for dampness pattern in AD and IBS-D, respectively, with the aim of identifying the accurate pattern-defined population of HXZQ. Master protocol is defined as a single overarching protocol designed with multiple substudies [21]. It allows the research questions to be addressed in an efficient way by minimizing time and infrastructure costs as compared with conducting separate studies [22]. Meanwhile, this study is actually developed under the pattern dominating different diseases research model (Yi Zheng Tong Bing model), which takes CM pattern as the target of intervention and focusses on the correlation between CM patterns and disease outcomes [23, 24]. Thus, the resulting procedures of our research will also provide a reference for future studies.

## 2. Methods

### 2.1. Objectives

The primary objective of this study is to evaluate the efficacy and safety of HXZQ oral liquid for dampness pattern in AD and IBS-D, respectively. Secondary objectives include (1) establishing two cohorts of AD and IBS-D to provide a study population basis and facilitate a long-term follow-up; (2) exploring the correlations of disease outcomes and dampness pattern, as well as other CM patterns; (3) developing a feasible study procedure of postmarketing evaluation for CM products under the pattern dominating different diseases research model.

### 2.2. Study Design and Setting

This master protocol consists of two multicenter, double-blinded, randomized placebo-controlled trials and a real-world observational study (Table 1): the CHARM trial (the Chinese herbal formula Huoxiang Zhengqi for atopic dermatitis with dampness pattern) [25] and the CHAIRS trial (the Chinese herbal formula Huoxiang Zhengqi for diarrhea-predominant irritable bowel syndrome) [26]. At the beginning of this trial, two registry-based cohorts of AD and IBS-D will be established, where patients with dampness pattern will be enrolled in the RCTs, and patients with nondampness pattern will be retained in the cohort constituting a real-world observational study. Thus, dampness pattern will be contrasted with nondampness pattern.

**Figure 1:**
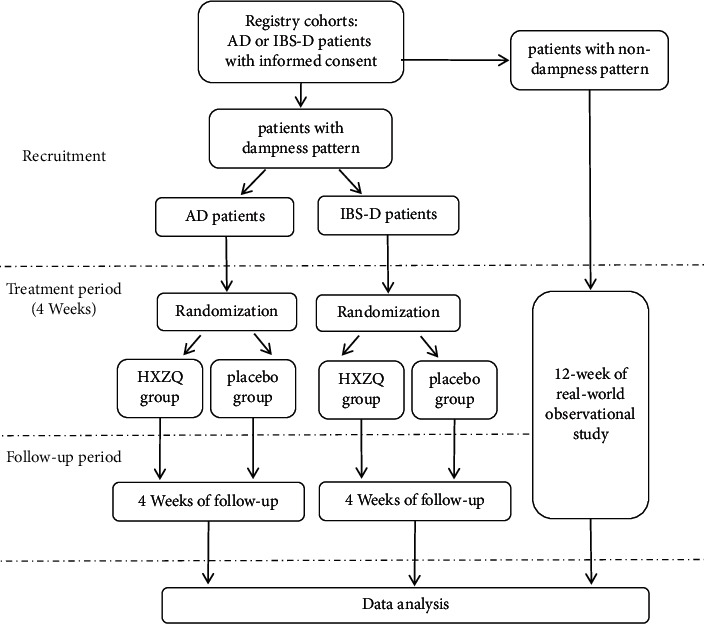
Study flowchart. AD, atopic dermatitis; IBS-D, diarrhea-predominant irritable bowel syndrome; HXZQ, Huoxiang Zhengqi oral liquid.

A total of 678 AD patients and 322 IBS-D patients will be enrolled from 14 hospitals in China over a 3-year period. Patients in the RCTs will be randomized at a ratio of 1 : 1 to receive either HXZQ oral liquid or a placebo for 4 weeks and then be followed up for another 4 weeks, while patients in the observational study will experience a 12-week follow-up.  Figure 1 displays the study flowchart. This study has been approved by the Ethics Committees of each participating site. Substudies have been registered in the Chinese Clinical Trial Registry (ChiCTR1900026700 and ChiCTR1900026837). An independent data safety monitoring board (DSMB) will monitor the overall conduct of the trial and assess the safety and efficacy of study procedures.

CHARM, Chinese herbal formula Huoxiang Zhengqi for atopic dermatitis with dampness pattern; CHAIRS, Chinese herbal formula Huoxiang Zhengqi for diarrhea-predominant irritable bowel syndrome; AD, atopic dermatitis; IBS-D, diarrhea-predominant irritable bowel syndrome; HXZQ, Huoxiang Zhengqi oral liquid.

### 2.3. Participants

#### 2.3.1. Recruitment

Participants will be recruited via posters in hospitals and advertisements in social media. To ensure a feasible and efficient recruitment, two registry-based cohorts will be established at the beginning of this trial. Based on predefined diagnostic criteria [6, 27], patients who present to a participating clinical site for treatment of AD will be screened for the AD cohort, and patients with IBS-D will be screened for the IBS-D cohort. In this registry period, broad eligibility criteria will be used to expedite the screening process. Then, eligible patients in the cohorts will further be classified as either “the dampness pattern” or “the nondampness pattern” following CM pattern differentiation. Subsequently, patients with dampness pattern will enter one of the RCTs, while patients with nondampness pattern will participate in the observational study. To be eligible for the RCTs, patients must again be screened against the specific inclusion and exclusion criteria (Table 2). Screening and enrollment logs will record enrolled patients, the reasons for exclusion, and the reasons eligible patients who are not enrolled.

#### 2.3.2. Inclusion Criteria of the Disease Cohorts

1. Patients between 18 and 70 years of age;
2. Informed consent obtained.

#### 2.3.3. Exclusion Criteria of the Disease Cohorts

1. Women who are pregnant, or lactating, or planning pregnancy;
2. Those who are currently enrolled in a study or had participated in a clinical trial within the past three months (one month for AD patients);
3. Anyone allergic to any medicine or ingredients used in the study;
4. Those who are considered unsuitable for the study by investigators.

#### 2.3.4. CM Pattern Differentiation

Patients in the disease cohorts will be classified as either dampness pattern or nondampness pattern depending on their clinical manifestations. Qualified CM clinicians will perform the pattern differentiation following relevant criteria [28]. Patients will be diagnosed as dampness pattern when meeting two main symptoms and one minor symptom of the general manifestations, or with one main symptom and two disease-specific manifestations. Otherwise, the nondampness pattern diagnosis will be made.

(1) General manifestations of dampness pattern
  - Main symptoms: mental fatigue; anorexia; loose stool or watery diarrhea; white sticky or greasy tongue coating.
  - Minor symptoms: heavy sensation in limbs; abdominal distension; pale tongue or pale teeth-marked tongue; soggy pulse or moderate pulse.
(2) Disease-specific manifestations of dampness pattern
  - AD: papules, papulovesicle, or blisters scattered over the limbs or other body parts; skin itching; white tongue coating.
  - IBS-D: loose stool or watery diarrhea; abdominal pain; white sticky greasy tongue coating; weak pulse.

### 2.4. Interventions

#### 2.4.1. Study Drug

In the two RCTs for AD and IBS-D, the experimental group will receive HXZQ oral liquid (20 ml, twice a day, for 4 weeks), and the control group will take the matched placebo (20 ml, twice a day, for 4 weeks). The HXZQ oral liquid and placebo will be manufactured by Taiji Group Chongqing Fuling Pharmaceutical Co. Ltd. (Chongqing, China) in accordance with the requirements of China good manufacturing practice (GMP) guidance. The placebo will be similar to HXZQ regarding appearance, weight, taste, and packaging. Compliance will be assessed upon a review of the bottles returned by patients.

#### 2.4.2. Concomitant Medications

For AD patients in the RCT, topical daily use of moisturizers such as urea ointment is recommended and permitted. Cetirizine hydrochloride tablets will be used in case of intolerable itching, and the dosage and frequency should be recorded. Other effective medications (orally or externally) and phototherapy for AD are not allowed. For IBS-D patients enrolled in the RCT, intolerable abdominal pain or diarrhea can be treated with emergency medications prescribed by the clinician. The name and dosage of the medication should be recorded in the patient's diary. Besides, any other medications and CM physiotherapy (such as acupuncture, moxibustion, or cupping) that may be effective for IBS-D, or can relieve abdominal pain and diarrhea, are prohibited. Treatments in the observational study will be provided by clinicians according to patients' specific conditions, and medical records of each patient should be exactly documented.

#### 2.4.3. Randomization and Blinding

Eligible patients in each RCT will be equally randomized to receive either HXZQ oral liquid or placebo through an interactive web response system. A center-stratified random sequence will be created with SAS 9.2 (SAS Institute Inc., Cary, USA). The randomization will be independently performed and managed by the Institute of Basic Research in Clinical Medicine (IBRCM), China Academy of Chinese Medical Sciences (Beijing, China). Patients and researchers involved in the trial including clinicians, research assistants, outcome assessors, and statisticians will be blinded to treatment allocation. In the observational study part, patients will receive no experimental interventions of this study. Investigators will just document the medical records, and blinding is not required.

### 2.5. Outcome Measurements

#### 2.5.1. Primary Outcome

For all AD patients, the primary outcome is the change in the Eczema Area and Severity Index (EASI) scores from baseline to the end of treatment (4^th^ week). EASI is the core outcome measurement instrument for AD, and the higher the score is, the more severe the AD is [29]. For IBS-D patients, the proportion of responders for adequate relief (AR) is the primary outcome evaluating the degree of symptom alleviation [30]. Throughout the study, IBS-D patients will be weekly asked the question: “Has your irritable bowel syndrome pain and discomfort adequately relieved in the past week?” (yes/no). Responders are defined as those who answer “yes” for at least 50% of the time, for example, in the RCT, that is, 2 out of 4 weeks in the treatment period. The proportion will be separately analyzed in the treatment period and the follow-up period.

#### 2.5.2. Secondary Outcomes

The secondary outcomes for AD include numerical rating scale (NRS) in the pruritus, investigator's global assessment (IGA) scale, body surface area (BSA), Skindex-29 (a dermatology-specific quality of life instrument), EuroQol-5-Dimensions-5-Level (EQ-5D-5L), antihistamine use, and CM pattern. Besides, EASI-50 and EASI-75 will be assessed, which refer to an EASI score showing improvement exceeding 50% or 75% during the treatment period, respectively [25]. For IBS-D patients, secondary outcomes are the Irritable Bowel Syndromes Symptom Severity Score (IBS-SSS), the Irritable Bowel Syndrome-Quality of Life Questionnaire (IBS-QOL), EQ-5D-5L, CM pattern manifestations [26].

### 2.6. Safety Assessment

Adverse events (AEs) will be reported to the investigators following the ICH-E2A guideline [31], and the causality between AEs and interventions will be assessed according to the WHO-UMC System for Standardized Case Causality Assessment [32]. Any event classified as “severe” or “life-threatening” in severity is considered a serious AE (SAE) and must be reported to the research ethics committee within 24 hours. Safety laboratory tests (routine blood, routine urinalysis, biochemical indexes of liver, and renal function) and electrocardiogram examination will be regularly conducted at baseline and 4^th^ week in the RCTs. Abnormal changes in laboratory examination will be judged by clinicians whether they have clinical importance and can be considered an AE. Safety assessment in the observational study will follow the guidance of clinicians.

### 2.7. Data Collection and Quality Control

A series of standard operating procedures will be established for data collection and quality control in the trial. Investigators and research assistants will receive a minimum of 4 hours training on study procedures prior to the enrollment. Detailed descriptions of the data collection schedule are provided in Table 3. Clinical data will be collected by a case report form (CRF) and then be transcribed into an electronic data capture (EDC) system developed by IBRCM. With EDC's consistency checking program and manual verification, all participant data will undergo validation checks for completeness, accuracy, and consistency.

The site principal investigator will oversee and be responsible for data collection, recording, and quality. The safety, integrity, and conduct of the trial will be monitored by an independent DSMB consisting of seven independent members. Besides, data monitoring will be entrusted to the Beijing Yaohai Ningkang Pharmaceutical Technology Co. Ltd. The auditing of the trial will be conducted by the Scientific Research Department at the Guangdong Provincial Hospital of Chinese Medicine, as well as the Office of the National Key Research and Development Program of China.

### 2.8. Statistical Analysis

#### 2.8.1. Sample Size Calculation

Sample size calculation of this trial consists of two parts: the RCTs and the observational study. Firstly, the sample size of each RCT was estimated, and the detailed estimation process can be found in subtrial protocols [25, 26]. In brief, a total of 430 patients diagnosed with dampness pattern (218 AD patients and 212 IBS-D patients) will be enrolled in the RCTs. Secondly, we assumed that the prevalence of dampness pattern is 39% in the AD population and is 81% within the IBS-D based on previous studies [33, 34]. Thus, 560 patients are required for the AD cohort and 270 for the IBS-D cohort. Considering a 15% loss to follow-up rate, the total sample size of cohorts is adjusted to 1000. Therefore, 570 patients diagnosed with nondampness pattern are required in the observational studies (as shown in Table 1).

#### 2.8.2. Statistical Methods

A statistical analysis plan (SAP) will be developed prior to the completion of the trial. All data will be analyzed by statisticians using SPSS 18.0 (IBM SPSS Inc., Armonk, New York, USA) and SAS 9.2 (SAS Institute Inc., Cary, USA), and two-sided *P* values <0.05 are considered statistically significant.

First, the analysis will be undertaken independently for each RCT [25, 26]. Baseline characteristics will be summarized using descriptive statistics and will be listed by groups. Efficacy analysis will be performed in both intention-to-treat (ITT) and per-protocol (PP) populations. Missing data will be processed with the multiple imputation method, and if necessary, sensitivity analysis will be used to explore the robustness of the results. For all outcomes, *t*-test or Mann–Whitney *U* test will be employed for continuous variables; Chi-squared test or Fisher's exact test will be used for categorical variables. The changes across time among outcomes will be analyzed by repeated measure analysis of variance. Center effect and other factors based on professional judgement like gender, age, and disease course will be adjusted by logistic regression model or Cox regression model. For safety assessments, comparisons of the incidence and severity of AEs or adverse drug reactions (ADRs) between the groups will be performed by Chi-square test, Fisher's exact test, or Wilcoxon rank-sum tests based on the data characteristics. Occurrences of AEs will be listed in a summary table containing the time of occurrence, severity, duration, adopted measures, causality assessment, and prognosis.

Second, the analysis of the observational study will be conducted for each of the two disease (AD and IBS-D) populations. To investigate the real-world based effectiveness in this part, some exploratory analysis methods will be adopted [35]. Briefly, baseline characteristics of these nondampness pattern patients will be described using univariate analysis to profile the general demographics, disease features, and medication uses for each disease group. Then, all outcomes will be analyzed and compared between different medication groups by propensity score matching (PSM). Safety assessments of observational part will follow the same procedures and methods as those in the RCTs.

Finally, an overall analysis will be performed combining multiple subgroups to comprehensively investigate the effect of HXZQ for dampness pattern. These subgroups analyses will include (1) HXZQ vs. placebo for AD patients, (2) HXZQ vs. placebo for IBS-D patients, (3) AD patients with dampness pattern vs. AD patients without dampness pattern, (4) IBS-D patients with dampness pattern vs. IBS-D patients without dampness pattern, and (5) patients with dampness pattern vs. patients without dampness pattern. We will pay special attention to the analysis of CM pattern, including the clinical manifestations, tongue and pulse signs, and pattern diagnosis. Based on the time sequence of clinical symptoms and CM pattern transition at each visit, a Markov model will be constructed to describe the changes of CM pattern over time [36]. The influence of interventions and related covariates (gender, age, disease, condition, CM pattern, etc.) on this change process will also be detected using mixed-effects models, or multilevel models. Then, to identify whether there are common or disease-specific changes of dampness pattern in different diseases, we will compare the dampness pattern changes of AD and IBS-D patients. Furthermore, the correlation of CM pattern and disease outcomes will be analyzed. Several methods such as logistic regression model, Cox regression model, and Bayesian hierarchical model will be explored and compared to establish a reasonable and accurate disease prognosis predictive model based on CM pattern [35].

## 3. Discussion

This master protocol was designed to evaluate the Chinese herbal formula HXZQ as a common dampness-resolving therapy for both AD and IBS-D patients. Combining the RCT and observational study design, this study can depict a more comprehensive efficacy profile of HXZQ through multiple subgroup comparisons, for example, patients with AD vs. patients with IBS-D, and patients with dampness pattern vs. patients without dampness pattern. Besides, the analysis of CM pattern changes in the two RCTs may enable us to identify the pattern-defined population of HXZQ that is with the best therapeutic effect. To our knowledge, a master protocol design has not been used to conduct efficacy research of HXZQ concurrently in multiple diseases, and its use will increase efficiencies in study design, participant enrollment, and data collection.

A master protocol is designed to answer several research questions within the same overall trial structure [21]. The general idea of the master protocol is to provide a platform for simultaneously evaluating multiple interventions [37]. It may involve one or more interventions in multiple diseases, or a single disease with multiple interventions [22]. This design concept is compatible with the flexibility of treatment based on pattern differentiation. In particular, when master protocol defines substudies based on CM pattern to investigate a common pattern-resolving therapy in different diseases, it is highly consistent with the CM therapeutic principle of “treating different diseases with the same therapy” [38]. Hence, master protocol design may facilitate the development of efficacy evaluation methods in CM. At present, there is also a growing interest in performing master protocol to study the efficacy of CM [39, 40].

In addition, different from previous studies that evaluate CM efficacy mainly on a disease basis, namely, disease dominating CM pattern research model (Yi Bing Tong Zheng model), we conduct this overarching trial from a pattern perspective. Participants are grouped by different CM patterns to enter either the RCTs or the observational study. The efficacy analysis will also be performed between different CM pattern groups. Importantly, the changes of CM pattern over time as well as the correlation of CM patterns and disease outcomes will be explored using multiple methods. Such a strategy is defined as CM pattern dominating different diseases (Yi Zheng Tong Bing model), closely reflecting the idea of “treating different diseases with the same therapy.” Compared with disease dominating pattern, the mainstream model in CM research, pattern dominating disease takes CM pattern as the target of intervention and evaluation, providing a parallel model for CM efficacy studies [24, 39]. To date, this model is still under development, and many efforts are needed to establish a feasible research framework, so it is also our hope that the resulting procedure in this study can provide a reference for future studies.

It should be mentioned that this study faces several challenges. First, the master protocol design increases coordination to bring all the stakeholders into agreement on trial design, conduct, and governance and adds to the difficulty of trial management as well as data analysis [22, 37]. To manage these complexities, we have had intensive pretrial discussions among sponsor and investigators to develop a more reasonable overall plan. Experts in the field have been invited to help with the study design and statistical analysis, and an independent DSMB was established to monitor the conduct of the study. Second, the classification of dampness pattern and nondampness pattern may be challenging. CM pattern differentiation is a fundamental procedure in our study determining which substudy a patient can be enrolled in. However, no reliable and accurate diagnostic criteria for dampness pattern have been developed, so the accuracy of pattern classification remains controversial. In response to this problem, we have conducted a literature survey to find a relatively credible standard and invited clinical experts to discuss and define a final criterion. Moreover, investigators were required to receive a training on criterion application. Third, the primary outcome is different in AD and IBS-D, making it difficult to directly compare the therapeutic effect on dampness pattern among multiple subpopulations. In general, the master protocol is designed on the basis of a common denominator, and its substudies often adopt the same outcome measurement to achieve efficacy comparability. Due to the lack of consistent criteria for efficacy evaluation on CM pattern [41, 42], we still choose the commonly recommended EASI score and AR responder proportion as the primary outcome [29, 30, 43]. The secondary outcomes of AD in our study include EASI-50 and EASI-75, which refer to an EASI score showing improvement exceeding 50% and 75%, respectively. To some extent, these two measurements have a similar explanatory effect to AR responder proportion, so the analysis and comparison of different subpopulations data could also be performed. Besides, other common outcome measurements such as EQ-5D-5L may also facilitate the comparison.

In conclusion, this study will provide high-quality efficacy evidence of HXZQ for AD and IBS-D patients and give an example of postmarketing evaluation for CM products under the pattern dominating different diseases research model.

## Figures and Tables

**Table 1:** Summary of study populations and intervention.

Trial name	Study design	Population	Intervention	Sample size
CHARM	RCT	AD with dampness pattern	HXZQ oral liquid vs. placebo	218
CHAIRS	RCT	IBS-D with dampness pattern	HXZQ oral liquid vs. placebo	212
—	Observational study	AD with nondampness pattern	—	460
		IBS-D with nondampness pattern		110

**Table 2:** Inclusion and exclusion criteria for patient selection in the RCTs.

Inclusion criteria	Exclusion criteria
Patients who meet all of the following criteria are eligible for the RCTs:	Patients who meet any of the following criteria are not eligible to participate in the RCTs:
1. Patients between 18 and 70 years of age and informed consent obtained	1. Patients with any uncontrolled cardiovascular, respiratory, digestive, urinary, or hematological disease; known cancer or mental illness
2. Diagnosed with CM dampness pattern	2. Examination results show ALT or AST levels exceeding 2 times the upper limit normal value; or BUN level exceeds 1.5 times the upper limit normal value during the screening period
3. CHARM (a) 1% < body surface area (BSA) < 10% (b) 1 ≤ investigator's global assessment (IGA) ≤ 3	3. Anyone allergic to any medicine or ingredients used in the study
	4. Women who are pregnant or lactating or plan to become pregnant in three months
4. CHAIRS (a) Initial irritable bowel syndromes symptom severity (IBS-SSS) score >75 (b) Patients had to be literate	5. Those who are considered unfit for the trial
	6. CHARM (a) those who have ever used HXZQ for treating eczema (b) medication used: systemic therapy with immunomodulatory or immunosuppressive agents (including tripterygium wilfordii) within the previous 4 weeks; topical or systematic therapy with antibiotics or antihistamines within the previous 2 weeks phototherapy (such as PUVA, UVA, or UVB) within the previous 4 weeks; topical therapy with immunomodulator within the previous 2 weeks; or topical or systematic use of any over-the-counter drugs within the previous week (c) those who are participating in any other clinical trial or had participated in a clinical trial within the previous month
	7. CHAIRS (a) Patients with warning signs (weight loss within 3 months of emaciation >10%), hematochezia that is confirmed not to have been caused by hemorrhoids or anal fissure, diarrhea at night, fever, family history of colorectal cancer (or polyposis syndrome), inflammatory bowel disease (IBD) or celiac disease (b) Organic diseases of the digestive system, or systemic diseases that affect digestive tract dynamics (c) Patients with a history of abdominal surgery (except caesarean section) (d) Those who are unwilling or unable to stop using drugs that affect the evaluation of interventions during the study (e) Standard points on the self-rating anxiety scale (SAS) > 50; standard points on the self-rating depression scale (SDS) > 53 (f) Those who have participated in other trials within the past 3 months

CHARM, Chinese herbal formula Huoxiang Zhengqi for atopic dermatitis with dampness pattern; CHAIRS, Chinese herbal formula Huoxiang Zhengqi for diarrhea-predominant irritable bowel syndrome; CM, Chinese Medicine.

**Table 3:** Study schedule.

		RCTs				Observational studies			
		Baseline	Treatment period		Follow-up	Baseline	Follow-up		
		0 week	2^nd^ week (AD only)	4^th^ week	8^th^ week	0 week	4^th^ week	8^th^ week	12^th^ week
Patient	Informed consent	×				×			
	Demographics	×				×			
	Medical history	×				×			
	Clinical manifestations	×		×	×	×	×	×	×
	CM pattern differentiation	×		×	×	×	×	×	×

Interventions	Randomization	×							
	HXZQ or placebo		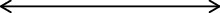						
	Medical record					×	×	×	×
	Concomitant medications		×	×	×				

Outcome measurements									
AD patients (only)	EASI	×	×	×	×	×	×	×	×
	BSA	×	×	×	×	×	×	×	×
	NRS、 IGA	×	×	×	×	×	×	×	×
	Skindex-29、EQ-5L-5D	×	×	×	×	×	×	×	×
	EASI-50、EASI-75		×	×	×		×	×	×
IBS-Dpatients (only)	Adequate relieve		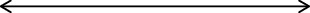				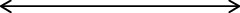		
	IBS-SSS	×		×	×	×	×	×	×
	IBS-QOL	×		×	×	×	×	×	×
	EQ-5D-5L	×		×	×	×	×	×	×

Safety assessment	Vital signs	×		×	×	×	×	×	×
	Laboratory tests and ECG	×		×		×			
	Adverse events		×	×	×		×	×	×

EASI, Eczema area and severity index; BSA, body surface area; NRS, numerical rating scale; IGA, investigator's global assessment; Skindex-29, a dermatology-specific quality of life instrument; EQ-5D-5L, euroQol-5-dimensions-5-level; IBS-SSS, irritable bowel syndromes symptom severity score; IBS-QOL, the irritable bowel syndrome-quality of life questionnaire.

## Data Availability

Data sharing of this paper is not applicable as no databases were generated at the current stage. Once the study is completed, data can be obtained from the corresponding author upon request.

## Abbreviations

AD: Atopic dermatitis
IBS-D: Diarrhea-predominant irritable bowel syndrome
CM: Chinese medicine
HXZQ: Huoxiang Zhengqi
DSMB: Data safety monitoring board
EASI: Eczema area and severity index scores
AR: Adequate relief
AE: Adverse event
ADR: Adverse drug reaction
EDC: Electronic data capture system.
